# Triclosan is associated with breast cancer via oxidative stress and relative telomere length

**DOI:** 10.3389/fpubh.2023.1163965

**Published:** 2023-05-05

**Authors:** Xiaomin Cai, Caibo Ning, Linyun Fan, Yanmin Li, Lu Wang, Heng He, Tianyi Dong, Yimin Cai, Ming Zhang, Zequn Lu, Can Chen, Ke Shi, Tianrun Ye, Rong Zhong, Jianbo Tian, Heng Li, Haijie Li, Ying Zhu, Xiaoping Miao

**Affiliations:** ^1^Department of Epidemiology and Biostatistics, School of Public Health, Tongji Medical College, Huazhong University of Science and Technology, Wuhan, China; ^2^Department of Epidemiology and Health Statistics, School of Public Health, Fujian Medical University, Fuzhou, China; ^3^Department of Occupational and Environmental Health, Key Laboratory of Environment and Health, Ministry of Education, School of Public Health, Tongji Medical College, Huazhong University of Science and Technology, Wuhan, China; ^4^Department of Urology, Tongji Hospital of Tongji Medical College, Huazhong University of Science and Technology, Wuhan, China; ^5^Department of Epidemiology and Biostatistics, School of Public Health, Wuhan University; Research Center of Public Health, Renmin Hospital of Wuhan University, Wuhan, China; ^6^Department of Gastrointestinal Cancer Research Institute, Tongji Hospital, Tongji Medical College, Huazhong University of Science and Technology, Wuhan, China

**Keywords:** TCS, BC, 8-OHdG, HNE-MA, 8-isoPGF_2α_, RTL, mediation effect

## Abstract

**Introduction:**

Triclosan (TCS), a widely prescribed broad-spectrum antibacterial agent, is an endocrine-disrupting chemical. The relationship and biological mechanisms between TCS exposure and breast cancer (BC) are disputed. We aimed to examine the correlation between urinary TCS exposure and BC risk and estimated the mediating effects of oxidative stress and relative telomere length (RTL) in the above association.

**Methods:**

This case-control study included 302 BC patients and 302 healthy individuals in Wuhan, China. We detected urinary TCS, three common oxidative stress biomarkers [8-hydroxy-2-deoxyguanosine (8-OHdG), 8-iso-prostaglandin F_2α_ (8-isoPGF_2α_), 4-hydroxy-2-nonenal-mercapturic acid (HNE-MA)], and RTL in peripheral blood mononuclear cells.

**Results:**

Significant associations were observed between log-transformed urinary concentrations of TCS, 8-OHdG, HNE-MA, 8-isoPGF_2α_, RTL, and BC risk, with the odds ratios (95% confidence intervals) being 1.58 (1.32–1.91), 3.08 (1.55–6.23), 3.39 (2.45–4.77), 3.99 (2.48–6.54), and 1.67 (1.35–2.09), respectively. Continuous TCS exposure was significantly positively correlated with RTL, HNE-MA, and 8-isoPGF_2α_ (all *p*<0.05) but not with 8-OHdG (*p* = 0.060) after adjusting for covariates. The mediated proportions of 8-isoPGF2_2α_ and RTL in the relationship between TCS and BC risk were 12.84% and 8.95%, respectively (all *p*<0.001).

**Discussion:**

In conclusion, our study provides epidemiological evidence to confirmed the deleterious effects of TCS on BC and indicated the mediating effect of oxidative stress and RTL on the correlation between TCS and BC risk. Moreover, exploring the contribution of TCS to BC can clarify the biological mechanisms of TCS exposure, provide new clues for the pathogenesis of BC, which is of great significance to improving public health systems.

## 1. Introduction

The latest statistics revealed that breast cancer (BC) is the most prevalent cancer (2,261,419 new cases per year) and ranked fifth in terms of cancer mortality worldwide in 2020, according to the International Agency for Research on Cancer (IARC) (1). In China, there are 0.42 million cases, accounting for approximately 18.3% of the global prevalence (1). Similar to the increase in incidence, increasing trends of mortality have also been observed in the past years, with approximately 117,174 deaths in China estimated to be 17.6% of the total deaths from BC (684,996 new deaths) (1). In the face of the rapidly increasing incidence and mortality, revealing the risk factors and etiology of BC is significant to guide the prevention, early screening, and treatment of tumors.

Previous research has uncovered definite risk factors that could partially explain the biological mechanisms of BC, such as intrinsic genetic susceptibility and extrinsic environmental exposure (2). With societal development, endocrine-disrupting chemicals (EDCs), which are potent and pervasive risks to human health, have attracted increasing attention recently, and may result in an incremental incidence of BC (3). Considering the ubiquitous presence of EDCs in products and the surrounding environment, investigation of EDCs exposure leading to BC is a priority.

Triclosan (TCS), a widely used broad-spectrum antibacterial agent, has attracted widespread attention because of its interference with the endocrine system or further cancer induction. Common TCS exposure routes from commercial and healthcare products include the skin and mucous membranes of the oral cavity and the gastrointestinal tract. Consequently, TCS can be measured in human biospecimens, such as urine, milk, and adipose tissue (4). As a type of EDCs, TCS was found to be associated with numerous adverse effects such as disruption of hormone homeostasis, inflammation, neuronal and cardiovascular damage, and carcinogenesis (5–8). Several studies in cell lines have indicated that TCS affects the incidence and progression of BC, but epidemiological studies have shown inconsistent results (9, 10). Therefore, it is worth investigating whether TCS plays a role in BC initiation and progression and exploring the possible mechanisms by which TCS induces BC.

Based on previous studies on the effect of TCS on tumorigenesis and development, it has been suggested that TCS is metabolized in the liver by cytochrome P450 (CYP), and this process involves a large number of metabolic processes, such as oxidative stress, cell proliferation, and immune response (4, 11). To date, exposure to TCS has been reported to promote ROS generation and induce oxidative stress (12–14), with oxidative stress makers significantly increasing in response to TCS treatment (15). Previous evidence has demonstrated that oxidative stress might facilitate the incidence and progression of BC (16–19). There was also evidence of a connection between fetal TCS exposure and relative telomere length (RTL) at birth (20). Additionally, by preventing end-to-end fusion, DNA double-strand breaks, nucleolytic decay, and degradation (21), telomeres play a crucial role in regulating cell reproduction and contribute to increasing mutations and susceptibility to cancer (22–25). Meanwhile, a prospective cohort analysis found longer telomeres to be risk factors for BC (26). Hence, the speculation on potential carcinogenic mechanisms of TCS is reasonable to perform that oxidative stress and telomere length might act as a mediator during the development of BC. However, to date, there are few population data intended to investigate their correlations among TCS exposure, BC risk, and the two indicators as intermediate variables, so the above conjecture provides new ideas and directions to elucidate the molecular mechanisms.

In the current study, we determined TCS, three biomarkers of oxidative stress [8-hydroxy-2-deoxyguanosine (8-OHdG), 8-iso- prostaglandin F_2α_ (8-isoPGF_2α_), and 4-hydroxy-2-nonenal-mercapturic acid (HNE-MA)] in urine, and relative RTL in peripheral blood mononuclear cells. We also estimated the associations between urinary TCS exposure and BC risk and further evaluated the underlying mediating effects of oxidative stress and RTL on the association between TCS and BC risk.

## 2. Materials and methods

### 2.1. Study population

We conducted a case-control study among 302 female patients with BC and 302 age-matched healthy controls. Participants were recruited between November 2016 and October 2019 at the Tongji Hospital of Huazhong University of Science and Technology, Wuhan, China.

The cases were newly diagnosed as primary BC by pathological confirmation, excluding cases with a BC family history, metastatic tumor from other organs, recurrent cancer, multiple primary tumors, or previous radiotherapy and chemotherapy treatment. The controls were healthy woman recruited from the Health Physical Examination Center of the same hospital and ensured no history of malignancy, no history of breast related benign tumors, no radiotherapy or chemotherapy. They are matched according to age within a range of ±5 years with the cases.

The specimen of study population included 2 ml of peripheral venous blood and 5 ml of morning urine from the study population before receiving radiotherapy and chemotherapy. While the demographic and clinical data were collected through the electronic medical record system.

### 2.2. Measure of urinary triclosan

#### 2.2.1. Chemicals and reagents

Triclosan and its internal standard (TCS-D3) were obtained from Toronto Research Chemicals (TRC, Toronto, Canada), methanol was obtained from Sigma-Aldrich (St. Louis, MO, US), purified water was obtained from a Milli-Q system (Millipore, Bedford, MA, US), and *β*-glucuronidase (from *Escherichia coli* K12) was obtained from Roche Diagnostics (Mannheim, Germany).

#### 2.2.2. Sample preparation and measure

According to previous studies with appropriate modifications, we prepared samples by liquid–liquid extraction (27). Briefly, TCS and TCS-D3 were prepared at a final concentration of 100 ng/ml, respectively, which were stored at −20°C until use. The calibration standard curve was diluted with fresh purified water to a concentration range of 0.0625–100 ng/ml. Urine specimens (500 μl) were digested with a *β*-glucuronidase solution (10 μl) for 12 h at 37°C and then centrifuged at 14,600 × *g* for 10 min. After centrifugation, the supernatant was added with working internal standard solution (50 μl) and then mixed for 30 s. Next, the mixture was added to ethyl acetate (3 ml) and sonicated for 60 min, then centrifuged at 3,000 × *g* for 10 min. The supernatant was then transferred to a gentle nitrogen stream at 35°C for drying. Finally, 50% (v/v) methanol (250 μl), which reconstituted the dried samples, was measured by ultra-high-performance liquid chromatography-high-resolution mass spectrometry (UHPLC-HRMS).

In order to ensure the accuracy and precision of the results from UHPLC-HRMS test, standard curve and linear range, intra−/inter-batch precision, recovery rate and the limit of detection (LOD) were used for quality control (QC). The specific criteria of detection were as follows:

(1) The linear range of measurement was evaluated by the concentration gradient of standard, and the coefficient of determination (R^2^) of the standard curve was greater than 0.997. If the concentration of TCS in the urine sample is much higher than the linear range of the calibration curve, re-analysis is performed by diluting the previous remaining sample to ensure the accuracy of the measurement; (2) The intra-batch precision was evaluated by analyzing three repeated samples in one day to ensure the intra-batch precision of TCS was less than 10%; (3) Inter-batch precision was evaluated by analyzing five urine samples from different batches with the same concentration to ensure the inter-batch precision was also less than 10%; (4) By mixing low and high concentration of standard solutions into 100 randomly selected urine samples, the recovery rate of different batches of TCS was in a range of 80 to 120%; (5) The reagent blanks (distilled water) are used to examine for contamination during the experiment; reagent blank TCS concentrations were lower than the LOD.

The results showed that the detection rate of TCS was 88.0%, and the LOD for TCS was 0.12 ng/ml. Meanwhile, the data below LOD was used LOD/√2 to instead of the original concentration. The recovery rate of TCS was in the range of 94.50–113.50%, and the intra-batch and inter-batch precision are 6.3% and 7.1%. Validated urine creatinine levels measured using commercial test kits were used to calibrate urine TCS concentrations, expressed as μg/g creatinine.

### 2.3. Measure of urinary oxidative stress biomarkers

#### 2.3.1. Chemicals and reagents

8-OHdG and HPLC-grade methanol were obtained from Sigma-Aldrich (St. Louis, MO, USA), HNE-MA was obtained from Zzstandard (Shanghai Zzbio Co. Ltd., Shanghai, China), the internal standards 15 N5-8-OHdG (25 μg/ml in water, 98% purity) and HME-NA-d3 (>98% purity) were obtained from Cambridge Isotope Laboratories (Andover, MA, USA), and 8-isoPGF_2α_ and its internal standard 8-isoPGF_2α_-d4 were obtained from Cayman Chemicals (Ann Arbor, MI, USA). Purified water was obtained using a Milli-Q system (Millipore, Bedford, MA, USA). Ammonium acetate was purchased from Sinopharm Chemicals (Shanghai, China).

#### 2.3.2. Sample preparation and measure

The method used to measure the concentrations of 8-OHdG, 8-isoPGF_2α_ and HNE-MA in urine by liquid chromatography–tandem mass spectrometry (LC–MS/MS), with some modifications, has been described in detail previously (28). The procedure and QC standard were similar to that used in a previous TCS assay. First, the sample was centrifuged at 10,000 rpm for 10 min and the supernatant (100 μl) was diluted with deionized water (1,500 μl). Second, the internal standard (50 μl) was added to the diluted sample for solid-phase extraction, the eluate was evaporated to dryness, and then redissolved in 5% methanol/water (200 μl). The target compounds were separated by high-performance liquid chromatography (HPLC) on a C18 column (3 μm, 100 × 2 mm Phenomenex Gemini-NX) and identified using an Agilent 6,460 triple quadrupole mass spectrometer. To assess the reliability of the analytical method, two quality controls and one blank sample from each batch were simultaneously measured. The results showed that the recoveries of the target compounds were in the range of 87.4–102.0%, and the intra-batch and inter-batch precision are 6.91% and 8.36%. The limits of qualification (LOQs) for 8-OHdG, 8-isoPGF_2α_ and HNE-MA were 0.08, 0.06 and 0.03 ng/ml, respectively.

### 2.4. Real-time quantitative PCR for relative telomere length

Relative telomere length, which was calculated by the copy-number ratio between telomeric repeats (T) and a single-copy (HGB) reference gene (S), was determined by Real-Time Quantitative PCR. The T/S ratio (ΔCt) is calculated from the mean telomere cycle threshold (Ct) value minus the mean HGB Ct value. ΔΔCt was calculated by subtracting the ΔCt of the standard sample from the ΔCt of each unknown sample, and then exponentiated (2^-ΔΔCt^). Briefly, DNA was isolated and quantified at 5.00 ng/μl an ultraviolet spectrophotometer. The telomere reaction mixture was consisted of 5.00 μl of Power SYBR Green Master Mix, 2.83 μl of RNase-free water, 1.00 μl of gDNA (5 ng/μl), 0.27 μl of Tel-Forward-[GGTTTTTGA (GGGTGA)_4_GGGT, 10 μmol/L], 0.90 μl of Tel-Reverse-[TCCCG AC(TATCCC)_5_TA, 10 μmol/L]. The HGB reaction consists of 5.00 μl of Power SYBR Green Master Mix, 3.60 μl of RNase-free water, 1.00 μl of gDNA (5 ng/μl), 0.20 μl of HGB-1-(GCTTCTGACACAACTG TGTTCACTAGC, 10 μmol/L), 0.90 μl of HGB-2-(CACCAAC TTCATCCACGTTCACC, 10 μmol/L). All primers were synthesized by Shanghai Shenggong Bioengineering Co. Ltd. The calibration standard curves ranged in concentration from 0.625 to 10 ng/μl. The flow of the reaction was first 1 cycle at 50°C for 2 min, followed by 40 cycles at 95°C for 3 s, and 60°C for 30 s. Finally, the melting curves were obtained at 95°C for 15 s, 60°C for 1 min, and 95°C for 15 s. To calculate the variability in inter- and intra-plate Ct values, quality control samples were scattered throughout the plates.

### 2.5. Statistical analyses

The concentrations of TCS, 8-OHdG, HNE-MA, 8-isoPGF_2α_ and RTL showed skewed distributions. Therefore, the log_10_-transformed TCS, 8-OHdG, HNE-MA, 8-isoPGF_2α_, and log_2_-transformed RTL were used for subsequent analyses. Demographic characteristics were partly described in a previous study (29). The various variables in our case-control study were appropriately analyzed using Student’s t-test, Chi-square test, Fisher test, and Mann–Whitney U test. Based on the median concentrations of urinary variables (TCS, 8-OHdG, 8-isoPGF_2α_ and HNE-MA) and RTL in the controls, we dichotomized all participants into low- and high-level groups. A logistic regression model was used to assess the associations between TCS, RTL, and oxidative stress biomarkers and BC with odds ratios (ORs) and 95% confidence intervals (CIs) after adjusting for age, smoking status, age at menarche, menopausal status, and abortion status, which have been proven to affect the development of breast cancer (30). Pearson correlation was used to study the relationship between TCS and indicators. Multiple linear regression and logistic regression models were used to estimate changes in RTL and oxidative stress biomarkers based on urine TCS levels using ORs derived from the beta coefficient (*β*).

To evaluate the role of oxidative stress biomarkers and RTL in mediating the correlation between TCS and BC, we used mediation analysis (31). Mediation analysis process using fitted linear regression and logistic regression models.


Y=βX+δC+ε(1)

$$M=\beta_{1}X+\delta_{1}C+\varepsilon_{1}\;(2)$$

$$Y=\beta_{2}X+\theta M+\delta_{2}C+\varepsilon_{2}\;(3)$$

$$Y=\beta_{3}X+\theta 'M+hX*M+\delta_{3}C+\varepsilon_{3}\;(4)$$

In the above formula, the variates *X*, *M*, and *Y* are the exposure to TCS, mediator, and outcome of BC, respectively, while *C* is a series of covariates. On the basis of the correlation between *X* and *Y* (Equation 1), the function—“mediate” in the package—“mediation” is used to analyze the mediating effect of the mediator *M* between *X* and *Y*. The parameter “model.m” is replaced by Equation 2, the parameter “model.y” is Equation 3, the parameter “treat” is the concentration of TCS, the parameter “mediator” is the concentration of each intermediate indicator. The remaining parameters are in the default format. Finally, the analysis outputs the average causal mediation effects (ACME), the average direct effects (ADE), and the proportions mediated (Prop. Mediated) to assess the mediated effects of oxidative stress markers and RTL. The ACME refers to the affection of TCS induces BC through mediating factors, which is complexly combined from Equation 2 and Equation 3, equaling *β*1 times θ. The ADE refers to the association between TCS and BC adjusted for the mediator, which means the effect is not influenced by mediators. It is equaled to the coefficient *β*2 in Equation 3. The coefficient *β* of Equation 1 represents the total effects, that is the influence of independent TCS exposure on BC before adding mediating variable, which consist of the direct effect and indirect effect. The Prop. Mediated refers to the proration of the ACME in the total effects, which represents the extent of mediated effects on the overall effect. Notably, it is necessary to conduct an interaction analysis between exposure and mediators by Equation 4 before mediation analysis. When there was a significant interaction, the parameter “model.y should use Equation 4 instead of Equation 3. Finally, since multiple mediators were of interest, a multiple mediation analysis was conducted by R package-“lavaan,” for exploring the possible effects between mediators on the association of TCS and BC (32, 33).

All statistical analyses were performed using Stata version 16 and R 4.0.3. Statistical significance was defined as a two-sided *p* < 0.05, except for partial threshold of *p*-values that were corrected by Bonferroni’s multiple hypothesis testing method, which has been indicated at the table and figure annotations.

## 3. Result

### 3.1. Characteristics of participants

The basic demographic characteristics of the study participants have been summarized in a previous study (29). In terms of the overall description, the case-control groups were well even in the distribution of age, smoking, menopausal status, and abortion status (all *p* > 0.05), except for age at menarche (*p* < 0.05).

In contrast, the distributions of urinary TCS, 8-OHdG, HNE-MA, 8-isoPGF2α, and RTL in the case-control study were uneven (Table 1). Notably, the median urinary TCS concentration of BC cases was 1.93 μg/g creatinine [interquartile range (IQR):0.45–11.38 μg/g creatinine], which was significantly higher than that of healthy controls (median:0.91 μg/g creatinine and IQR:0.28–3.07 μg/g creatinine; *p* < 0.001). Additionally, BC patients had a significantly longer RTL than the controls (*p* < 0.001). The median urinary oxidative stress biomarker concentrations (8-OHdG, HNE-MA, and 8-isoPGF_2α_) of BC cases were also significantly higher than those of controls (all *p* < 0.05).

**Table 1:** The distribution of urinary triclosan (TCS), 8-OHdG, HNE-MA, 8-isoPGF_2α_ and relative telomere length (RTL) in the case-control study.

	Cases	Controls	*P* ^b^
	(*n* = 302)	(*n* = 302)	
TCS^a^, μg/g, median (IQR)	1.93 (0.45, 11.38)	0.91 (0.28, 3.07)	**<0.001**
8-OHdG^a^, μg/g, median (IQR)	5.33 (3.61, 7.99)	4.64 (3.41, 6.40)	**0.002**
HNE-MA^a^, μg/g, median (IQR)	34.78 (12.01, 109.11)	13.48 (8.62, 27.89)	**<0.001**
8-isoPGF_2α_^a^, μg/g, median (IQR)	4.94 (2.97, 8.75)	3.41 (2.04, 5.39)	**<0.001**
RTL, median (IQR)	1.53 (1.07, 2.41)	1.34 (0.84, 2.01)	**<0.001**

a The urinary TCS, 8-OHdG, HNE-MA and 8-isoPGF_2α_ concentration was adjusted for creatinine.

b *p*-Value refers to Mann–Whitney *U*-test.

TCS, triclosan; 8-OHdG, 8-hydroxy-2-deoxyguanosine; 8-isoPGF_2α_, 8-iso-prostaglandin F_2α_; HNE-MA, 4-hydroxy-2-nonenal-mercapturic acid; RTL, relative telomere length; IQR, interquartile range.

### 3.2. Correlation of urinary TCS with the risk of BC

We observed significant associations between urinary TCS levels and BC risk in our study population (Table 2). With log_10_-transformed continuous-type urinary TCS concentrations, the elevated TCS dose per unit was attributed to a raised BC risk for 1.58-fold (95% CI:1.32–1.91, *p* < 0.001) after adjusting for age, smoking status, age at menarche, menopausal status, and abortion status. When the urinary TCS concentrations were classified into low- and high-exposure groups, individuals with a high TCS exposure level also had a higher BC risk than those with low TCS exposure (OR = 1.96, 95% CI = 1.40–2.74, *p* < 0.001). Notably, the stratified analysis showed that the risk of breast cancer was higher under certain conditions, such as age > 60 years, age at menarche >13 years, menopause status, and abortion status (Table 3).

**Table 2:** The association of urinary triclosan (TCS), 8-OHdG, HNE-MA, 8-isoPGF_2α_ and relative telomere length (RTL) with breast cancer (BC) risk.

	Cases	Controls	OR^b^ (95% CI)	*P* ^c^
	(*n* = 302)	(*n* = 302)		
TCS^a^				
Per ug/g unit increment			1.58 (1.32, 1.91)	**<0.001**
TCS categories, No. (%)				**<0.001**
Low	103 (34.1)	151 (50.0)	1.00 (ref.)	
High	199 (65.9)	151 (50.0)	1.96 (1.40, 2.74)	
Oxidative stress markers				
8-OHdG^a^				
Per ug/g unit increment			3.08 (1.55, 6.23)	**0.002**
8-OHdG categories, No. (%)				0.017
Low	124 (41.1)	151 (50.0)	1.00 (ref.)	
High	178 (58.9)	151 (50.0)	1.49 (1.07, 2.08)	
HNE-MA^a^				
Per ug/g unit increment			3.39 (2.45, 4.77)	**<0.001**
HNE-MA categories, No. (%)				**<0.001**
Low	81 (26.8)	151 (50.0)	1.00 (ref.)	
High	221 (73.2)	151 (50.0)	2.72 (1.94, 3.86)	
8-isoPGF_2α_^a^				
Per ug/g unit increment			3.99 (2.48, 6.54)	**<0.001**
8-isoPGF_2α_ categories, No. (%)				**<0.001**
Low	96 (31.8)	151 (50.0)	1.00 (ref.)	
High	206 (68.2)	151 (50.0)	2.19 (1.56, 3.09)	
RTL				
Per unit increment			1.67 (1.35, 2.09)	**<0.001**
RTL categories, No. (%)				0.041
Short	123 (40.7)	151 (50.0)	1.00 (ref.)	
Long	179 (59.3)	151 (50.0)	1.44 (1.02, 2.04)	

a The urinary TCS, 8-OHdG, HNE-MA and 8-isoPGF_2α_ concentration was adjusted for creatinine.

b The logistic regression model was adjusted for age, smoking status, age of menarche, menopausal status, abortion status.

c Statistical significance was corrected by Bonferroni’s multiple hypothesis testing method, and the bold values means there are significant difference.

Log_10_-transformed for TCS, 8-OHdG, HNE-MA and 8-isoPGF_2α_; Log_2_-transformed for RTL.

TCS, triclosan; 8-OHdG, 8-hydroxy-2-deoxyguanosine; 8-isoPGF_2α_, 8-iso-prostaglandin F_2α_; HNE-MA, 4-hydroxy-2-nonenal-mercapturic acid; RTL, relative telomere length; BC, breast cancer; OR, odds ratio; CI, confidence interval.

**Table 3:** Stratified analyses about the association between triclosan (TCS) and breast cancer (BC) by age, age at menarche, menopause status, and abortion status.

Groups	TCS^a^		*P* ^c^
	Low	High	
	OR^b^ (95% CI)	OR^b^ (95% CI)	
Age, years
≤60 (*N* = 494)	1.00 (ref.)	1.59 (1.10, 2.30)	**0.014**
>60 (*N* = 110)	1.00 (ref.)	5.74 (2.44, 14.44)	**<0.001**
Age at menarche, years
≤13 (*N* = 305)	1.00 (ref.)	1.14 (0.71, 1.83)	0.594
>13 (*N* = 299)	1.00 (ref.)	3.85 (2.32, 6.51)	**<0.001**
Menopause status
Yes (*N* = 223)	1.00 (ref.)	3.80 (2.14, 6.92)	**<0.001**
No (*N* = 381)	1.00 (ref.)	1.38 (0.91, 2.11)	0.130
Abortion status
Yes (*N* = 342)	1.00 (ref.)	2.34 (1.49, 3.71)	**<0.001**
No (*N* = 262)	1.00 (ref.)	1.57 (0.95, 2.61)	0.080

a The urinary TCS concentration was adjusted for creatinine.

b The logistic regression model was adjusted for age, smoking status, age of menarche, menopausal status, abortion status.

c Statistical significance was corrected by Bonferroni’s multiple hypothesis testing method, and the bold values means there are significant difference.

TCS, triclosan; BC, breast cancer; OR, odds ratio; CI, confidence interval.

### 3.3. Correlation of oxidative stress biomarkers and RTL with BC

The prevalence of BC tends to increase with a gradual increase in urinary oxidative stress biomarker concentrations (Table 2). The ORs (95% CI) of 8-OHdG, HNE-MA and 8-isoPGF_2α_ were 3.08 (1.55–6.23), 3.39 (2.45–4.77), 3.99 (2.48–6.54), respectively. To better understand the correlation between oxidative stress and BC, individuals were divided into low- and high-concentration groups according to biomarker concentrations. Compared with individuals in the low-concentration groups of the above oxidative stress biomarkers, individuals with a high concentration of HNE-MA, and 8-isoPGF_2α_ also had a higher risk of BC (Table 2). Moreover, significant positive associations were observed between BC risk and RTL (Table 2). Subsequently, we conducted stratified analyses according to age, age at menarche, menopausal status, and abortion status. We found that HNE-MA and 8-isoPGF_2α_ were significantly correlated with BC risk in all stratified groups, while 8-OHdG and RTL were associated with BC risk, especially in women aged over 60 years, with older age at menarche, menopausal status, or history of abortion (Supplementary Tables S1–4).

### 3.4. Estimated changes of oxidative stress biomarkers and RTL according to urine TCS levels

**Figure 1:**
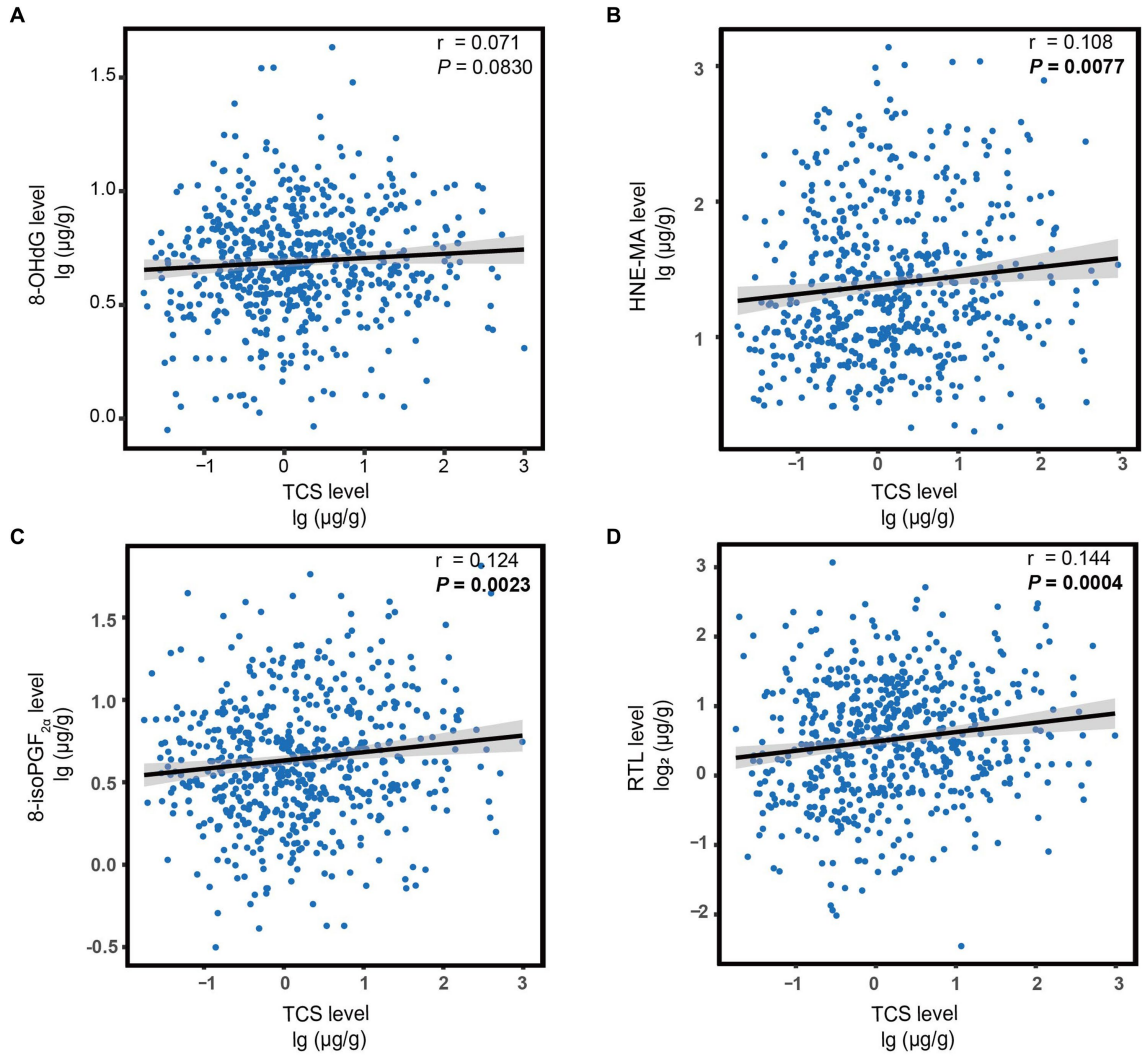
The correlation between urine TCS levels and oxidative stress biomarkers **(A–C)** or RTL **(D)**. Each figure depicts a regression line with 95% confidence intervals, and the circles represent individual data points. The *p*-values were calculated using Spearman’s correlation. Statistical significance was corrected by Bonferroni’s multiple hypothesis testing method. Log_10_-transformed for TCS, 8-OHdG, HNE-MA, and 8-isoPGF_2α_; Log_2_-transformed for RTL. TCS, triclosan; RTL, relative telomere length; 8-OHdG, 8-hydroxy-2-deoxyguanosine; HNE-MA, 4-hydroxy-2-nonenal-mercapturic acid; 8-isoPGF_2α_, 8-iso-prostaglandin F_2α_.

Next, we explored the correlations between urinary TCS and oxidative stress biomarkers and RTL. As expected, urinary TCS levels were positively correlated with oxidative stress biomarkers and RTL. Higher TCS exposure was associated with higher HNE-MA, higher 8-isoPGF_2α_, and longer RTL (all *r* > 0 and all *p* < 0.01; Figure 1). Therefore, in linear regression models adjusted for covariates, per unit raised of log_10_-transformed urinary TCS was corelated with a 0.06 and 0.05 unit raised in log_10_-transformed HNE-MA and 8-isoPGF_2α,_ respectively (both *p* < 0.013). However, we did not observe a significant relationship between TCS and 8-OHdG levels (*p* = 0.060). When changing the TCS and other indicators into categorical variables, the results showed similar trends. Although logistic regression analysis did not show a significant association between high-level TCS exposure and high-level 8-OHdG (*p* > 0.05), other oxidative stress biomarkers still indicated possible differences, reflected by a 1.52-fold or 1.43-fold higher risk compared to low-level exposure and responses. Furthermore, with the increase in each unit in the log_10_-transformed urinary TCS, the log_2_-transformed RTL increased by 0.11 (*p* = 0.001)_._ Likewise, there was an increase in the long RTL group compared to the short RTL group (Table 4).

**Table 4:** Estimated changes of urinary 8-OHdG, HNE-MA, 8-isoPGF_2α_ and relative telomere length (RTL) according to urine triclosan (TCS) levels.

	TCS^a^				
	*β*^b^ (95% CI)	*P* ^d^	OR^c^ (95% CI)		*P* ^d^
			Low	High	
8-OHdG^a^	0.02 (−0.001, 0.04)	0.060	1.00 (ref.)	1.28 (0.92, 1.79)	0.142
HNE-MA^a^	0.06 (0.01, 0.11)	0.013	1.00 (ref.)	1.52 (1.09, 2.13)	0.014
8-isoPGF_2α_^a^	0.05 (0.02, 0.08)	**0.003**	1.00 (ref.)	1.43 (1.02, 2.00)	0.038
RTL	0.11 (0.05, 0.18)	**0.001**	1.00 (ref.)	1.44 (1.02, 2.04)	0.041

a The urinary TCS, 8-OHdG, HNE-MA and 8-isoPGF_2α_ concentration was adjusted for creatinine.

b The linear regression model was adjusted for age, smoking status, age of menarche, menopausal status, abortion status.

c The logistic regression model was adjusted for age, smoking status, age of menarche, menopausal status, abortion status.

d Statistical significance was corrected by Bonferroni’s multiple hypothesis testing method, and the bold values means there are significant difference.

Log_10_-transformed for TCS, 8-OHdG, HNE-MA and 8-isoPGF_2α;_ Log_2_-transformed for RTL.

TCS, triclosan; 8-OHdG, 8-hydroxy-2-deoxyguanosine; 8-isoPGF_2α_, 8-iso-prostaglandin F_2α_; HNE-MA, 4-hydroxy-2-nonenal-mercapturic acid; RTL, relative telomere length; BC, breast cancer; OR, odds ratio; CI, confidence interval.

### 3.5. Mediating effects

**Figure 2:**
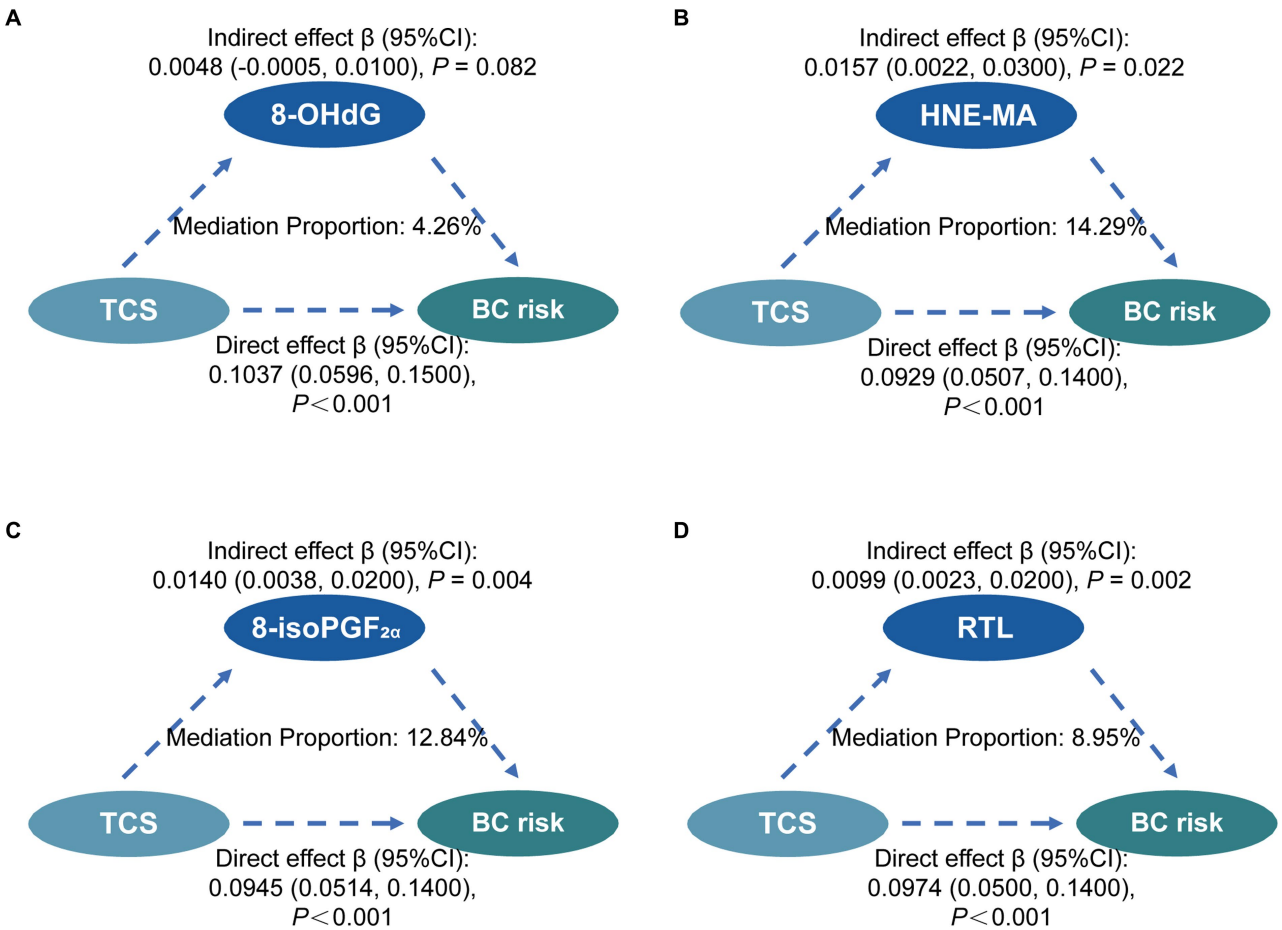
Mediation effects of oxidative stress and RTL on the associations between TCS exposure and the risk of breast cancer. Mediating effects of 8-OHdG **(A)**, HNE-MA **(B)**, 8-isoPGF_2α_ **(C)**, and RTL **(D)** on the association between TCS and BC risk. The direct effect, indirect effect, and mediation proportion were estimated using the R package mediation. The models were adjusted for urinary creatinine level, age, smoking status, age at menarche, menopausal status, and abortion status. Statistical significance was corrected by Bonferroni’s multiple hypothesis testing method. TCS, triclosan; RTL, relative telomere length; 8-OHdG, 8-hydroxy-2-deoxyguanosine; HNE-MA, 4-hydroxy-2-nonenal-mercapturic acid; 8-isoPGF_2α_, 8-iso-prostaglandin F_2α_; BC, breast cancer; CI, confidence interval.

In the multivariate-adjusted models, the levels of oxidative stress biomarkers and RTL were associated with both TCS exposure and BC risk. Hence, we conducted mediation analyses of oxidative stress biomarkers and RTL in relation to urinary TCS and BC risks (Figure 2). Before mediation analysis, the interaction analyses were conducted between exposure and mediators. The interaction analyses found there was no interactions between TCS and three indicators of oxidative stress except RTL (*β* = −0.28, 95%CI = −0.513—−0.051, *p*-value = 0.017).

In the mediation analyses, we found no mediation by 8-OHdG and HNE-MA in the association between urinary TCS and BC risk. However, it was significantly mediated by 8-isoPGF_2α_ and RTL in similar mediation models. The proportion of the relationship between urinary TCS and BC risk mediated in part by 8-isoPGF_2α_ and RTL was 12.84%, and 8.95%, respectively. Finally, the multiple mediation analysis shows no evidence of a significant relationship between 8-isoPGF_2α_ levels and RTL in the mediation of the association between TCS exposure and breast cancer risk (Supplementary Figure S1).

## 4. Discussion

Here, through an intensive study of BC patients and healthy controls, we found that higher TCS concentrations were associated with elevated BC risk. This association could be mediated partly by HNE-MA, 8-isoPGF_2α_ and RTL.

Environmental studies have demonstrated that phenols have a high detection rate in the body and can induce tumorous cellular proliferation (34). As a widely used phenol, TCS is distributed ubiquitously across the ecosystem, which means that paying attention to environmental exposure to TCS in the human body is necessary (35). Hence, we measured the concentration of TCS in urine, which reflects the exposure more directly and accurately than in the external environment, and found that TCS exposure was positively associated with BC progression. Several population-based studies have shown a strong association between triclosan exposure and the incidence of newly diagnosed breast cancer in women (36). The results of our population study are consistent with those of previous studies.

As exposure to TCS may promote the development of breast cancer, it is worth exploring potential biological mechanisms. Based on previous research, biochemical and molecular mechanisms are diverse, such as cellular longevity and oxidative stress (4). In this study, we found that TCS exposure was associated with longer telomere length and increased HNE-MA and 8-isoPGF_2α_, which represent lipid peroxidation. To date, only a few studies based on population data have elucidated the correlation between TCS exposure and oxidative stress or telomere length. Studies among Puerto Rican pregnant women, as well as among Chinese and Brazilian children suggested dose-dependent effects on urinary TCS levels and oxidative stress (13, 37, 38). While the Harvard Epigenetic birth cohort discovered a correlation between TCS exposure and telomere length (20), a similar correlation between EDCs and telomere length was also observed in some population studies (39, 40). Therefore, we further focused on these two aspects to explore the mediating effects of TCS and BC, as illustrated in our study.

Previous research has suggested that TCS has strong lipophilicity in organisms, indicating that its related biochemical metabolic pathways are involved in oxidative stress (41). Many studies have shown that oxidative stress produces excessive ROS and that free radicals can cause tissue damage and lead to BC development (42, 43). Identify with forward researches’ result, we found a positive association between oxidative stress and the prevalence of BC, as primarily manifested by increased levels of 8-OHdG, HNE-MA and 8-isoPGF_2α_, which was also found in our previous study (44, 45). Furthermore, the findings of our study confirmed that the lipid peroxidation biomarkers HNE-MA and 8-isoPGF_2α_ but not the DNA oxidative biomarker 8-OHdG, partly mediated the correlation between urinary TCS and BC. This suggests that lipid peroxidation is a critical intermediate biological pathway influencing TCS exposure on BC risk.

ROS-induced oxidative damage to DNA is positively associated with carcinogenesis, which inhibits or induces transcription, replication errors, genomic instability, and changes in telomere length (46, 47). Telomeres are considered an indicator of cell senescence and apoptosis, as they shorten with cell division (47). When the length of telomeres in normal cells is critically limited, an apoptotic mechanism can be triggered (48). Thus, maintaining telomere length is an essential process in cancer progression and a recognized hallmark of cancer (49). However, epidemiological studies on telomere length and risk of BC have shown inconsistent results. Two retrospective case-control studies and a prospective cohort study reported a positive association between telomere length and BC (26, 50, 51), whereas others found a negative or null association (52, 53). Our study provides more favorable data for significantly positively associated populations. Interestingly, mediation analysis demonstrated that the association between urinary TCS and BC was partly mediated by RTL, suggesting that RTL plays an important role in TCS-associated BC risk.

At present, there are few epidemiological studies on the relationship between TCS and BC, and no research has been devoted to illuminating the mechanism of RTL and oxidative stress caused by TCS in BC. Our research provides not only epidemiological evidence for a positive association between TCS exposure and BC prevalence, but also explains the mediators in the association. Moreover, exploring the contribution of TCS to BC can clarify the biological mechanisms of TCS exposure, provide new clues for the pathogenesis of BC, and identify high-risk populations, which is of great significance to improving public health systems. Nevertheless, our study has several limitations. First, our findings provide insufficient evidence of a causal association between urinary TCS and BC. Second, we only collected urine samples at one point in time, which may be less accurate than repeated measurements for a long-term exposure and could not avoid the nondifferential misclassification of exposure from the intraindividual variability. Third, the sample collection was less comprehensive and lacking of several important relevant data, such as BMI, which may reduce the reliability of research results. Therefore, our findings need to be validated in future prospective cohort studies with more detailed measurements.

## 5. Conclusion

In summary, our study found that urinary TCS was significantly associated with BC incidences, suggesting that TCS exposure increases the risk of BC. Further, we suggest that oxidative stress and telomere length might partially mediate the impact of TCS on the occurrence of BC.

## Data availability statement

The original contributions presented in the study are included in the article/Supplementary material, further inquiries can be directed to the corresponding authors.

## Ethics statement

The studies involving human participants were reviewed and approved by The ethics committee of Tongji Medical College of Huazhong University of Science and Technology. The patients/participants provided their written informed consent to participate in this study. Written informed consent was obtained from the individual(s) for the publication of any potentially identifiable images or data included in this article.

## Author contributions

XC and CN performed the statistical analysis and wrote the draft of the manuscript. XC, LF, LW, YL, CN, TD, YC, MZ, ZL, CC, KS, and TY contributed to investigation, resources. RZ, JT, HJL, YZ, and XM contributed to conception and design of the study. All authors contributed to manuscript revision, read, and approved the submitted version.

## Funding

This work was supported by National Key Research and Development Program of China (2022YFA0806600, 2022YFA0806601), National Science Fund for Distinguished Young Scholars of China (NSFC-81925032), Key Program of National Natural Science Foundation of China (NSFC-82130098) and Natural Science Foundation of Hubei Province (2019CFA009) for XM; Youth Program of National Natural Science Foundation of China (NSFC-82003547) for YZ; Youth Program of National Natural Science Foundation of China (NSFC-82103929) and Fundamental Research Funds for the Central Universities (2042022kf1205) for JT.

## Conflict of interest

The authors declare that the research was conducted in the absence of any commercial or financial relationships that could be construed as a potential conflict of interest.

## Publisher’s note

All claims expressed in this article are solely those of the authors and do not necessarily represent those of their affiliated organizations, or those of the publisher, the editors and the reviewers. Any product that may be evaluated in this article, or claim that may be made by its manufacturer, is not guaranteed or endorsed by the publisher.
