# Supplementary figures and images for: Triclosan is associated with breast cancer via oxidative stress and relative telomere length
Source: Front Public Health. 2023 May 5;11:1163965. doi: 10.3389/fpubh.2023.1163965 (PMC10197149; doi:10.3389/fpubh.2023.1163965)

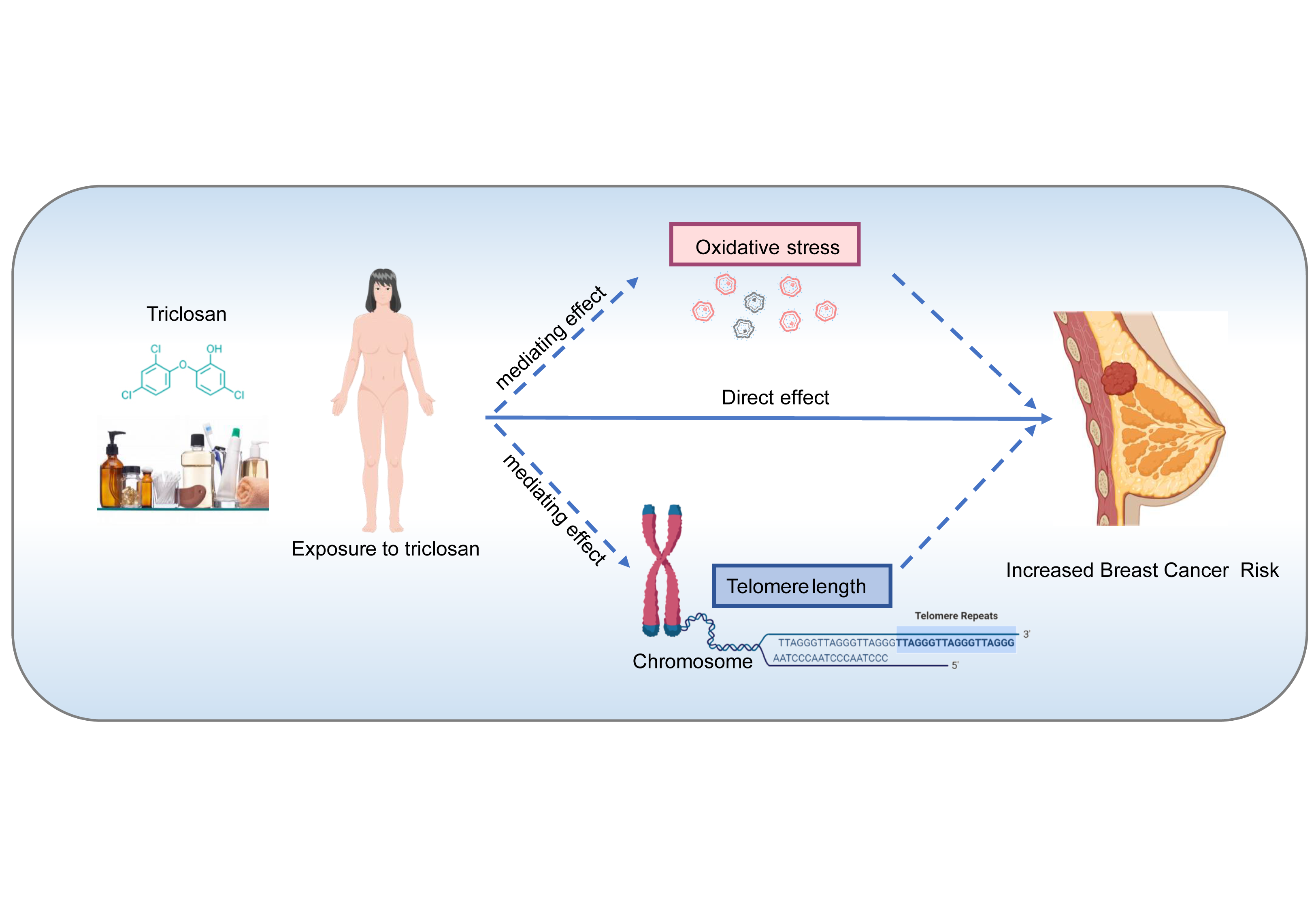

Supplement: Supplementary file 1 [file Image_1.TIF]

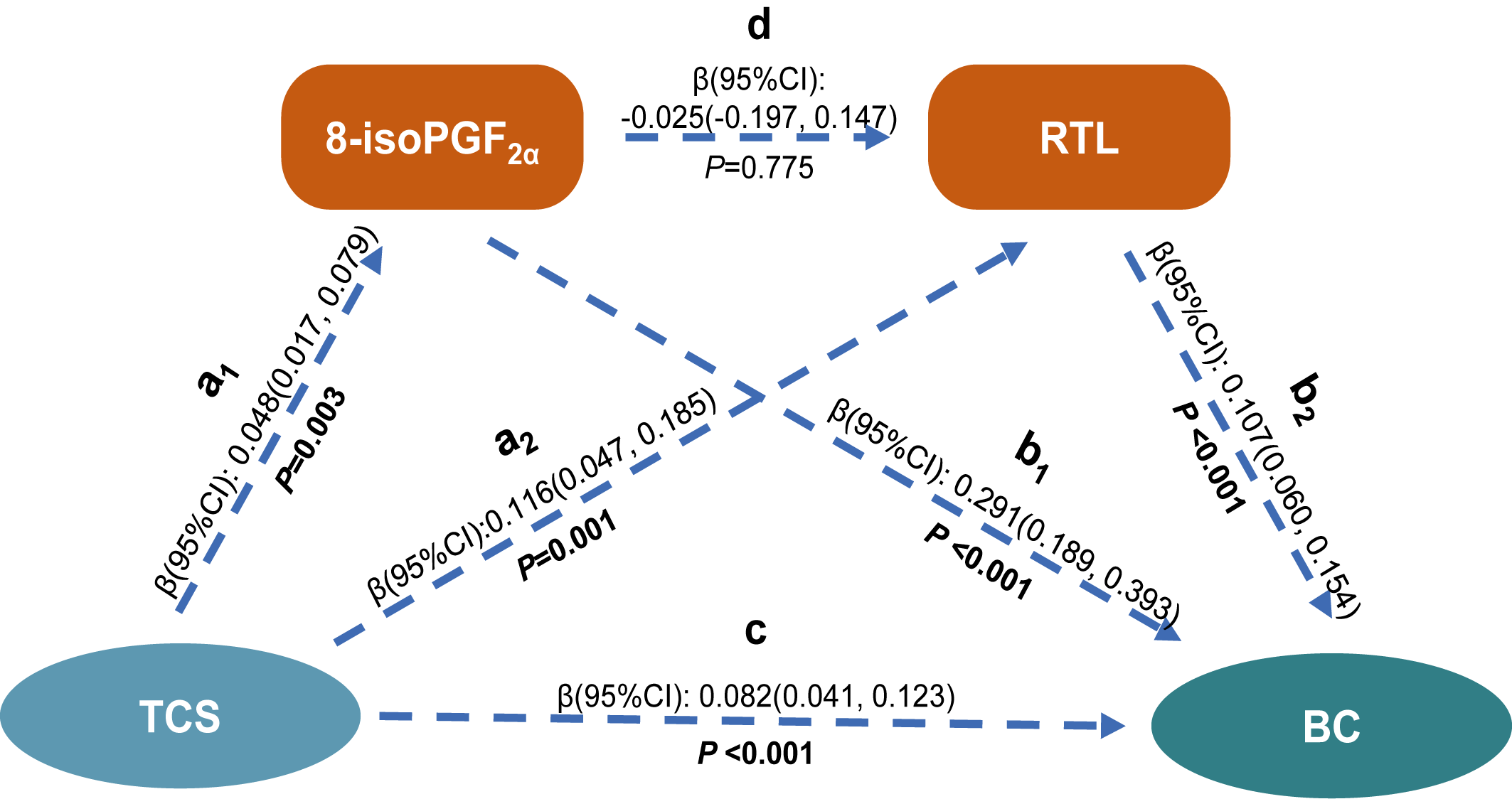

Supplement: Supplementary file 2 [file Image_2.TIF]
